# A review of the protection mechanisms of ginsenoside CK on cardiovascular disease and stroke

**DOI:** 10.3389/fphar.2026.1777145

**Published:** 2026-04-22

**Authors:** Hui-Ting Chan, Yu-Po Lee, Yu-Quan Chang, Lichieh Julie Chu, Tzu-Chun Tsai, Chin-Kuo Chen, Sheau-Long Lee, Guang-Huar Young, Yi-Huan Wu, Robert Y. L. Wang

**Affiliations:** 1 Biotechnology Industry Master and Ph.D. Program, Chang Gung University, Taoyuan, Taiwan; 2 Wellhead Biological Technology Corp., Taoyuan, Taiwan; 3 Graduate Institute of Biomedical Sciences, College of Medicine, Chang Gung University, Taoyuan, Taiwan; 4 Department of Medical Affairs & Planning, Taipei Veterans General Hospital, Taipei, Taiwan; 5 Department of Otolaryngology–Head and Neck Surgery, Chang Gung Memorial Hospital, Keelung, Taiwan; 6 Department of Biomedical Sciences, College of Medicine, Chang Gung University, Taoyuan, Taiwan

**Keywords:** antioxidant, cardiovascular disease, ginsenoside compound k, myocardial ischemia, stroke

## Abstract

Cardiovascular disease (CVD), comprising coronary artery disease, myocardial infarction, arrhythmias, heart failure, and hypertension, predominantly originates from dysfunction or obstruction within the cardiac and vascular systems. It has persistently remained the leading cause of death worldwide. The classification of stroke is based on its pathogenesis, with ischemic and hemorrhagic types representing distinct classifications. The etiology of stroke is attributed to cerebral vascular occlusion or rupture. It is frequently associated with hypoxic injury and neuronal necrosis, which poses a significant burden on individuals and public health systems. Ginsenoside compound K (CK), a secondary metabolite derived from the intestinal microbial transformation of protopanaxadiol type ginsenosides (e.g., Rb1, Rb2), exhibits high oral bioavailability and the capacity to cross the blood-brain barrier. Recent research has demonstrated that CK possesses antiplatelet and antithrombotic activities, inhibits vascular smooth muscle cell proliferation and endothelial inflammation, and shows low toxicity along with antiarrhythmic potential in the cardiovascular system. Furthermore, CK demonstrates significant anti-inflammatory, antioxidative, and mitochondrial protective properties, exhibiting efficacy in models of myocardial ischemia/reperfusion and cerebral ischemia. It exhibits good tolerability and safety without significant antagonism to other drugs and is increasingly recognized as a promising multi-target natural therapeutic candidate. The objective of this review is twofold: first, to synthesize recent findings on the mechanisms by which ginsenoside CK confers protection in cardiovascular and cerebrovascular conditions; and second, to assess its translational potential and highlight its prospective role in next-generation cardiovascular therapies.

## 1 Introduction

### 1.1 Global epidemiology of cardiovascular disease (CVD) and stroke

Cardiovascular disease (CVD) comprises a wide range of heart and vascular disorders, including coronary artery disease (e.g., myocardial infarction), arrhythmias, heart failure, peripheral arterial disease, and hypertension. As stated in the Global Burden of Disease Report 2020, CVD continues to be the foremost cause of mortality on a global scale, accounting for more than 17.9 million deaths on an annual basis, constituting approximately 32% of all global deaths (Roth et al., 2020). Ischemic heart disease and stroke account for the largest share of disability-adjusted life years (DALYs) worldwide (Roth et al., 2020; Yusuf et al., 2021). The pathophysiological basis of chronic CVD includes endothelial dysfunction, persistent low-grade inflammation, vascular remodeling, and oxidative stress. Collectively, these factors result in the cumulative impairment of cardiovascular and cerebrovascular integrity over time (Libby, 2006). A stroke is defined as an acute cerebrovascular event that can be categorized as either ischemic (∼85%) or hemorrhagic (∼15%). Ischemic strokes are caused by the occlusion of arteries, usually due to atherosclerosis or thrombosis. In contrast, hemorrhagic strokes are the result of vascular rupture and intracranial bleeding. According to the Lancet Neurology (2021), more than 12 million new strokes occur globally each year, with over 6 million resulting in death (Feigin et al., 2021). Moreover, it is imperative to acknowledge the profound challenges faced by over 100 million individuals who have survived strokes, as evidenced by the prevalence of chronic neurological or cognitive impairments in this demographic. This phenomenon exerts a substantial strain not only on caregivers but also on healthcare systems, underscoring the critical need for effective therapeutic interventions and comprehensive support services (GBD 2016 Stroke Collaborators, 2019). Although acute-phase interventions such as intravenous thrombolysis and mechanical thrombectomy have proven effective in revascularization, their capacity to facilitate neural regeneration and long-term recovery remains limited, highlighting the urgent need for novel, multi-phase therapeutic strategies (Moskowitz et al., 2010). Notwithstanding advances in pharmacotherapy (e.g., antiplatelets, thrombolytics) and interventional approaches (e.g., percutaneous coronary intervention [PCI]), persistent issues of endothelial dysfunction, oxidative stress, inflammation, and adverse vascular remodeling continue to compromise long-term outcomes (Kim, 2012).

### 1.2 The etiology, clinical manifestations, and preventive strategies for cardiovascular disease and stroke

Cardiovascular diseases (CVDs) and stroke represent a significant global burden, accounting for a considerable number of deaths and cases of disability. The pathogenesis of cardiovascular disease (CVD) is primarily associated with atherosclerosis, hypertension, diabetes mellitus, hyperlipidemia, and unhealthy lifestyle behaviors, including smoking, obesity, and a lack of physical activity. The broad classification of stroke encompasses two primary categories: ischemic and hemorrhagic. Ischemic stroke is typically caused by thrombotic or embolic occlusion of cerebral arteries, leading to interrupted cerebral perfusion. Conversely, hemorrhagic stroke is characterized by the rupture of cerebral vessels, resulting in intracranial bleeding. The common clinical manifestations of stroke include hemiparesis, aphasia, headache, and visual disturbances. Preventive strategies are centered on comprehensive management of major modifiable risk factors, including hypertension, hyperglycemia, and hyperlipidemia, alongside the promotion of regular exercise, a balanced diet, smoking cessation, alcohol moderation, and stress reduction. In addition, the early diagnosis of cardiovascular disease (CVD) and stroke, in conjunction with the provision of robust public health education, has been demonstrated to have a pivotal role in the reduction of both incidence and mortality rates (Lindsay et al., 2019).

### 1.3 Ginsenosides as multi-target natural therapeutics

The demand for multi-target therapies has spurred interest in naturally occurring bioactive compounds, especially ginsenosides—the major triterpenoid saponins derived from *Panax* species, including *Panax ginseng*, *Panax notoginseng*, and *Panax quinquefolium*. More than 60 structurally diverse ginsenosides have been identified to date, with the protopanaxadiol (PPD) and protopanaxatriol (PPT) subclasses showing significant cardioprotective and neuroprotective activities (Song et al., 2022).

### 1.4 Structural classification of ginsenosides and biotransformation to CK

**FIGURE 1:**
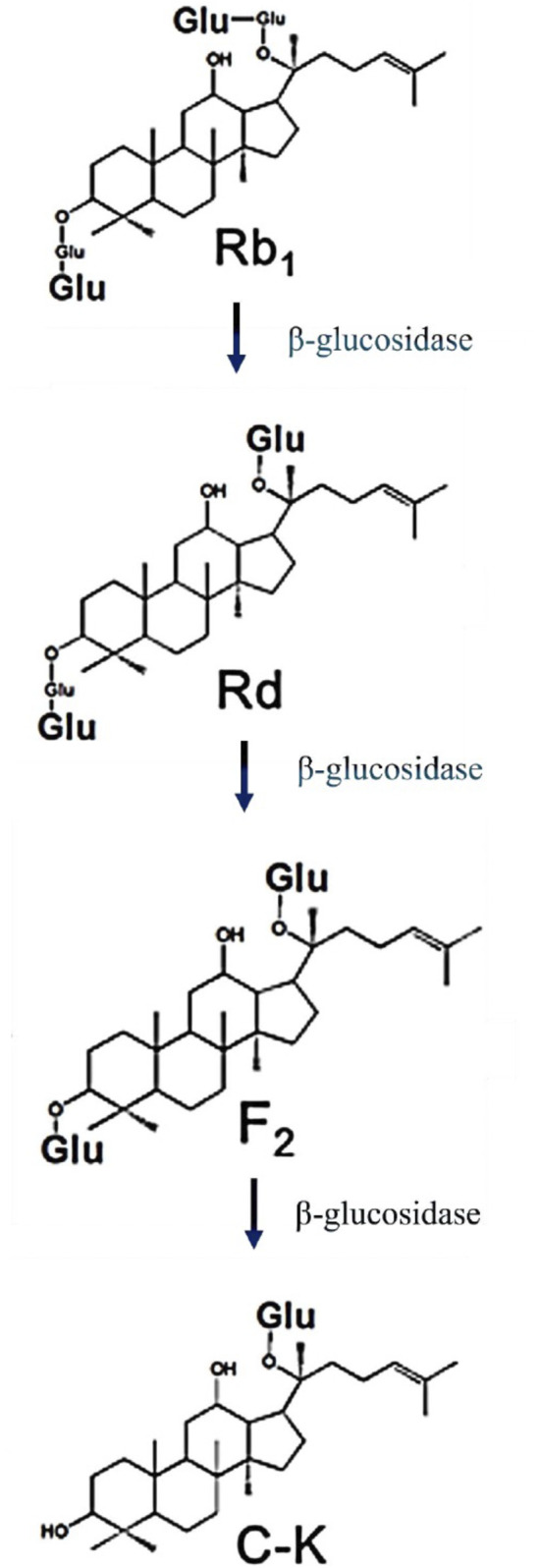
The conversion of protopanaxadiol-type ginsenosides to Compound . It is to be noted that Glu is Glucose.

Ginsenosides are broadly classified into two principal structural categories based on their aglycone skeleton: protopanaxadiol (PPD)-type and protopanaxatriol (PPT)-type. PPD-type ginsenosides encompass a group of compounds, including Rb1, Rb2, Rc, Rd, Rh2, and compound K (CK). These ginsenosides are distinguished by the presence of sugar moieties attached at the C-3 and/or C-20 positions of the molecular structure. Conversely, PPT-type ginsenosides, including Rg1, Re, Rf, and Rh1, exhibit glycosylation predominantly at the C-6 position. Ginsenoside compound K (CK) is a deglycosylated secondary metabolite formed by the gut microbiota through the conversion of PPD-type ginsenosides (e.g., Rb1, Rb2, Rc). The biotransformation pathway is illustrated in (Figure 1) (Morshed et al., 2024). It involves the following stages: Rb1 → Rd→F2→CK, primarily mediated by microbial β-glucosidase. Key microbial genera involved include *Bacteroides* and *Eubacterium* (Zheng et al., 2021). Industrially, CK can be synthesized via microbial fermentation using *Aspergillus niger* (Hasegawa et al., 1996) or enzymatically via immobilized recombinant β-glucosidase hydrolysis (Tian et al., 2022).

CK is not a naturally occurring ginsenoside found in high concentrations in unprocessed in raw *Panax* species but rather, it is a product of complex process involving intestinal microbial deglycosylation of PPD-type ginsenosides. The canonical biotransformation pathway proceeds as follows: Rb1 → Rd → F2 → CK. It is important to note that PPT-type ginsenosides do not serve as direct precursors of CK. The structural relationship under discussion is this structural configuration elucidates the reason for CK retaining the PPD aglycone backbone while exhibiting reduced polarity and enhanced membrane permeability in comparison with its parent compounds.

The following methods have been demonstrated to be effective in the preparation and manufacturing of CK (Hu et al., 2023). *In vivo* microbial biotransformation: CK is generated through a series of steps involving the removal of glucose molecules from protopanaxadiol-type ginsenosides (Rb1 → Rd → F2 → CK). This process is facilitated by the actions of gut microbiota, particularly those genera that produce β-glucosidase, such as *Bacteroides* and *Eubacterium* (Morshed et al., 2024). Industrial microbial fermentation. The implementation of controlled fermentation using *A. niger* or engineered microbial strains enables scalable production with improved yield and purity (Chen et al., 2018). Enzymatic biocatalysis: Immobilized recombinant β-glucosidases have been shown to facilitate regioselective hydrolysis with a high degree of specificity, thus constituting a reliable pharmaceutical-grade production strategy (Chen et al., 2022). Semi-synthetic approaches: Selective hydrolysis and structural modification have been explored; however, biotransformation remains the dominant approach.

The molecular formula of CK is C_36_H_62_O_8_, characterized by low polarity, enabling efficient membrane permeability and penetration of the blood–brain barrier (BBB). It demonstrates high oral bioavailability, a relatively long half-life (6–8 h), and favorable tissue distribution. Mechanistically, CK modulates multiple signaling cascades, such as *PI3K/Akt*, *NF-κ B*, *AMPK–mTOR*, and *Nrf2/Keap1* to elicit antioxidant, anti-inflammatory, anti-apoptotic, and metabolic regulatory effects, exerting therapeutic benefits in both cardiovascular and neurological disease models (Chen et al., 2022; Chen et al., 2024; Ding et al., 2022; Liu J. et al., 2022; Wang et al., 2022) (Table 1). Collectively, these properties position CK as a potent multi-target therapeutic candidate, especially for managing chronic cardiocerebrovascular disorders.

**TABLE 1:** Systemic targets and pathways of Ginsenoside CK.

Pathway	Major target proteins	Functional implication
*PI3K–Akt*	*AKT1*, PDK1, FOXO3	Cell survival, metabolic regulation, neuroprotection
*NF-κBNF-κB*	RELA, NFKB1, IκBα	Inflammation, immune regulation
JAK–STATJAK	STAT3, IL6, JAK2	Immune homeostasis, tumor suppression
MAPK	MAPK3, ERK1/2	Vascular remodeling, neuronal plasticity
Apoptosis	TP53, *BAX*, BCL2	Anti-cancer, anti-stroke potential
Angiogenesis	*VEGFA*, *HIF-1α*	Cardioprotection, post-stroke recovery
Neurodegeneration	GPX4, ACSL4	CK recently shown to inhibit ferroptosis in aging brain

### 1.5 The blood-brain barrier permeability and neuroprotective potential of ginsenoside CK

Emerging evidence indicates that ginsenoside CK is capable of crossing the blood–brain barrier (BBB) and exerting therapeutic effects within the central nervous system, thereby supporting its potential for management of stroke and neurodegenerative disorders. CK has been demonstrated to enhance neurological recovery in models of ischemic stroke by modulating the *Nrf2/ARE* antioxidant pathway, reducing ROS production, stabilizing mitochondrial membrane potential (MMP), and inhibiting neuronal apoptosis. It is important to note that CK exerts dual regulatory effects on vascular endothelial cells and glial cells, thereby mitigating neuroinflammation and preserving BBB integrity following stroke (Zhao et al., 2022). Its lipophilicity and low molecular weight facilitate both active transport and passive diffusion across the BBB. Furthermore, interaction with ABC transporters (e.g., P-gp) has been demonstrated to promote CK accumulation in the brain. CK has also been demonstrated to exhibit affinity for Alzheimer’s disease-associated proteins (e.g., Aβ and Tau), with studies indicating its ability to reduce neuroinflammation, oxidative stress, and synaptic damage, thus highlighting its potential to prevent post-stroke neurodegenerative processes (Li et al., 2022). In animal models of Alzheimer’s disease, CK has been found to suppress microglial overactivation and IL-1β expression, restore synaptic protein expression, and enhance memory performance through BBB repair (Wu et al., 2023). These findings provide support for the multifaceted neuroprotective roles of CK in chronic post-stroke conditions involving complex pathological mechanisms.

## 2 Mechanisms of action of ginsenoside CK in cardiovascular disease and stroke

Ginsenoside CK, a deglycosylated metabolite of protopanaxadiol-type ginsenosides, has been the subject of extensive research due to its multifaceted therapeutic actions in cardiovascular and cerebrovascular diseases. While a considerable number of ginsenosides have been the subject of exploration, the present section focuses on the distinct mechanisms by which CK exerts its effects. The mechanisms in question are classified into ten principal pathways, as delineated in Table 2 and discussed below.

**TABLE 2:** Summary Table of Ginsenoside CK experimental models and actions.

Types of treatment	Section no./Test model	Function/Mode of action	Cell line/Test dosage	IC50 (CK only)
Cardiomyocyte ischemia/reperfusion	2.1/*in vivo* (rat I/R model)	Activates *PI3K*–*Akt*; inhibits ROS and autophagy	H9c2 cells/5–20 μM	17.2 μM (H9c2)
Myocardial contractility preservation	2.2/*ex vivo* heart perfusion	Enhances SOD, GPx, reduces Ca^2+^ overload	HL-1 cells/10 μM	N/A
Pulmonary arterial hypertension	2.3/monocrotaline rat model	Inhibits *NF-κB/NLRP3*; reduces PASMC proliferation	PASMCs/10–30 μM	24.5 μM (PASMCs)
VSMC proliferation inhibition	2.4/PDGF-BB-induced rat model	Arrests G1 phase, suppresses MMP-2, -9	A7r5/10 μM	20.4 μM
Endothelial protection (ox-LDL)	2.5/HUVECs	Suppresses *NF-κB*, *p38*, *JNK*	HUVEC/10 μM	12.3 μM
CAM expression suppression	2.6/TNF-α-stimulated model	Inhibits *VCAM-1*, *ICAM-1*, E-selectin	HUVEC/5–20 μM	15.1 μM
Neuroprotection in ischemia	2.7/OGD/R SH-SY5Y	Regulates *AMPK/mTOR*; reduces apoptosis	SH-SY5Y/10 μM	18.9 μM
Mitochondrial dynamics (cerebral I/R)	2.8/MCAO rat model	Preserves Mfn2; inhibits Mul1–*DRP1* axis	Neuronal primary culture/10–25 μM	21.7 μM
Neurogenesis post-ICH	2.9/thrombin-induced NSC damage	Activates *LXRα*; upregulates *HMGB3*, *RBBP7*	NSCs/10 μM	N/A
Microglia inflammation suppression	2.10/LPS-activated BV2 model	Downregulates *NF-κB*; increases *HO-1*	BV2/10–30 μM	16.8 μM

### 2.1 Protection against myocardial ischemia/reperfusion injury via PI3K-Akt pathway

**FIGURE 2:**
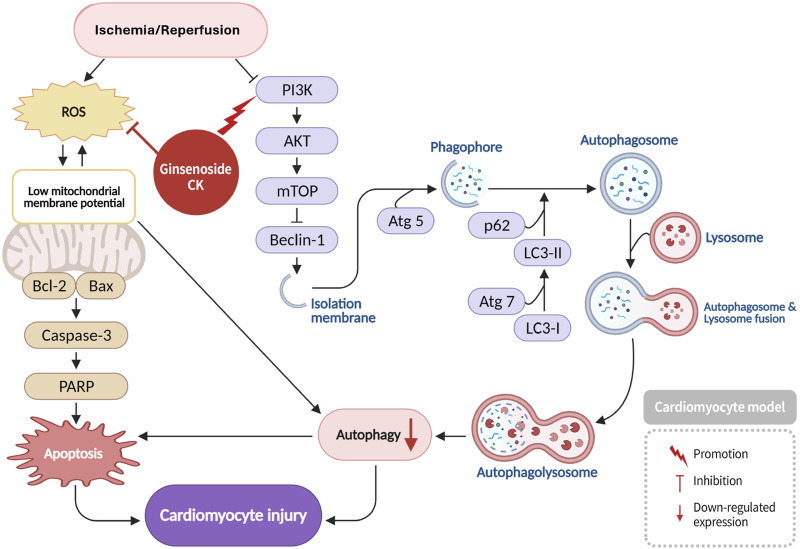
Protective mechanism of Ginsenoside CK against myocardial ischemia/reperfusion injury via PI3K Akt pathway activation and caspase-3 inhibition.

Ischemia/reperfusion (I/R) injury to the heart has been demonstrated to result in impaired phosphoinositide 3-kinase (PI3K) signaling pathway activity in cardiomyocytes, consequently inducing the initiation of downstream autophagy processes. Concurrently, I/R injury has been demonstrated to elevate the production of reactive oxygen species (ROS), increase mitochondrial membrane permeability, and disrupt mitochondrial membrane potential (Hu et al., 2023). Ginsenoside CK confers cardioprotection during ischemia/reperfusion (I/R) injury by modulating autophagy-mediated apoptosis. I/R injury impairs the PI3K-Akt signaling axis, leading to increased autophagy, excessive reactive oxygen species (ROS) production, mitochondrial membrane permeabilization, and release of pro-apoptotic mediators. CK activates PI3K-Akt signaling, inhibits autophagic flux, reduces ROS accumulation, and prevents cardiomyocyte death, thereby preserving myocardial integrity (Figure 2) (Li et al., 2018).

### 2.2 Preservation of myocardial contractility post-ischemia

**FIGURE 3:**
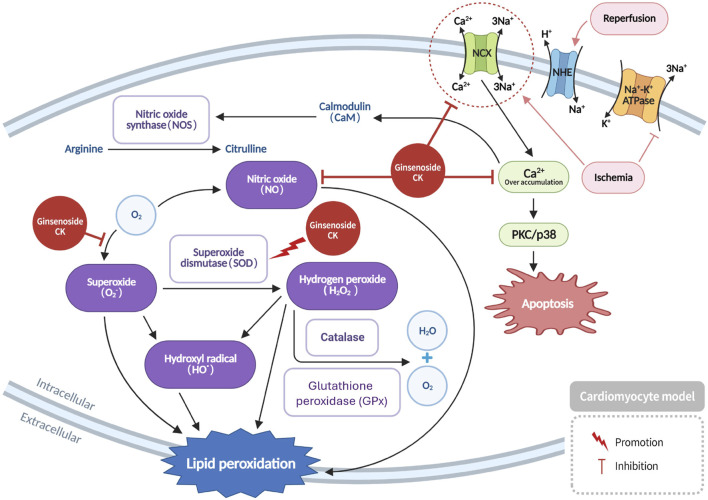
Restoration of myocardial contractility by CK through modulation of intracellular calcium homeostasis and reduction of ROS generation following ischemia.

Myocardial ischemia leads to a significant reduction in oxygen and nutrient supply to cardiomyocytes, resulting in cellular damage and the excessive generation of reactive oxygen species (ROS) (Kim, 2007; Kim, 2009), including superoxide anions, hydroxyl radicals, hydrogen peroxide, and nitric oxide. These ROS interact with cellular membranes, initiating lipid peroxidation and thereby disrupting membrane integrity and damaging both cells and surrounding tissue. Furthermore, the process of ischemia/reperfusion (I/R) injury has been demonstrated to induce a dysregulation of ion homeostasis by means of stimulation of the Na^+^/H^+^ exchanger (NHE), with the resultant effect being an intracellular sodium (Na^+^) overload. This effect is further compounded by the inhibition of the Na^+^/K^+^-ATPase pump, thereby impairing the cell’s capacity to extrude excess Na^+^ (Kim, 2007; Yellon and Hausenloy, 2007). In response, cardiomyocytes activate the Na^+^/Ca^2+^ exchanger (NCX) to restore sodium balance. However, this can inadvertently increase intracellular calcium (Ca^2+^) levels, contributing to impaired myocardial contractility and calcium overload injury. As indicated by recent studies, ginsenoside CK has been demonstrated to mitigate oxidative stress induced by myocardial ischemia by reducing ROS accumulation and enhancing the activity of antioxidant enzymes such as superoxide dismutase (SOD), catalase, and glutathione peroxidase (GPx) (Figure 3) (Kim, 2007; Kim, 2009). In addition, evidence has emerged demonstrating the capacity of CK to impede the deleterious influx of Ca^2+^ during reperfusion, thereby safeguarding the functionality of cardiomyocytes and exerting substantial cardioprotective effects (Kim, 2007; Kim, 2009) (Figure 3).

Mechanistically, CK-mediated preservation of myocardial contractility involves stabilization of intracellular calcium homeostasis. CK has been demonstrated to modulate Na+/Ca^2+^ exchanger activity and attenuate reperfusion-induced Ca^2+^ overload. This, in turn, prevents mitochondrial permeability transition pore (mPTP) opening and subsequent apoptotic signaling.

### 2.3 Amelioration of pulmonary arterial hypertension via inhibition of NF-κB/NLRP3 inflammasome signaling

**FIGURE 4:**
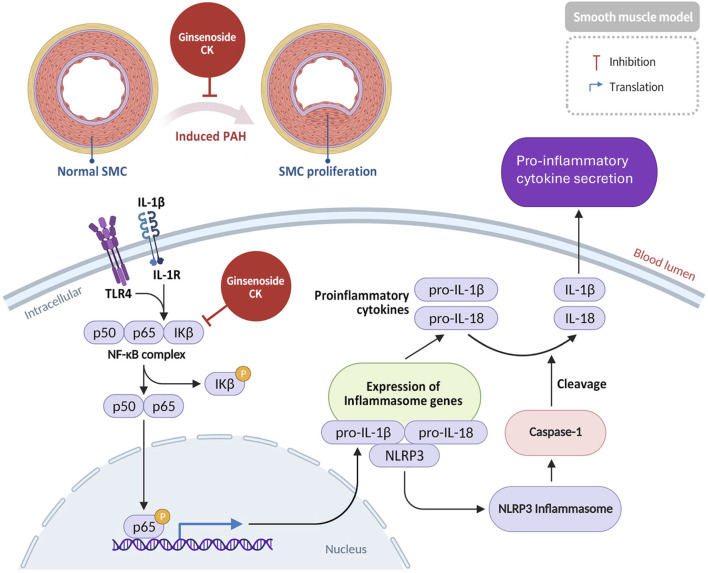
Amelioration of pulmonary arterial hypertension by CK via inhibition of NF-κB/NLRP3 inflammasome signaling and vascular remodeling.

Pulmonary arterial hypertension (PAH) is a potentially lethal condition marked by persistent elevation in mean pulmonary arterial pressure, which fosters pulmonary vascular remodeling and eventually results in right heart failure (Lan et al., 2018). A hallmark of this pathological process is the abnormal proliferation of pulmonary arterial smooth muscle cells (PASMCs) and perivascular inflammation, both of which contribute significantly to pulmonary vascular remodeling (Tian et al., 2013). Recent studies have demonstrated the efficacy of ginsenoside CK in suppressing proliferation of pulmonary arterial smooth muscle cells (PASMC) in rat models of pulmonary arterial hypertension (PAH). Furthermore, CK has been shown to inhibit activation of the NF-κB/NLRP3 signaling axis and inflammasome formation within pulmonary tissue, leading to a marked reduction in the expression of key pro-inflammatory cytokines, including IL-1β and IL-18 (Zhu et al., 2026) (Figure 4). The present findings support the hypothesis that ginsenoside CK has therapeutic potential as a candidate for the prevention and treatment of pulmonary arterial hypertension. This phenomenon can be attributed to its capacity to target both vascular remodeling and inflammatory pathways in a concomitant manner (Huang et al., 2021).

At the molecular level, CK exerts its function by suppressing *NF-κB* nuclear translocation and inhibiting NLRP3 inflammasome assembly by reducing ROS-dependent priming signals. Downstream suppression of caspase-1 activation limits IL-1β and IL-18 maturation, thereby attenuating pulmonary vascular inflammation and smooth muscle proliferation. These effects suggest that CK interferes with both the inflammatory initiation and amplification phases in PAH pathogenesis.

### 2.4 The inhibition of vascular smooth muscle cell proliferation and intimal hyperplasia

**FIGURE 5:**
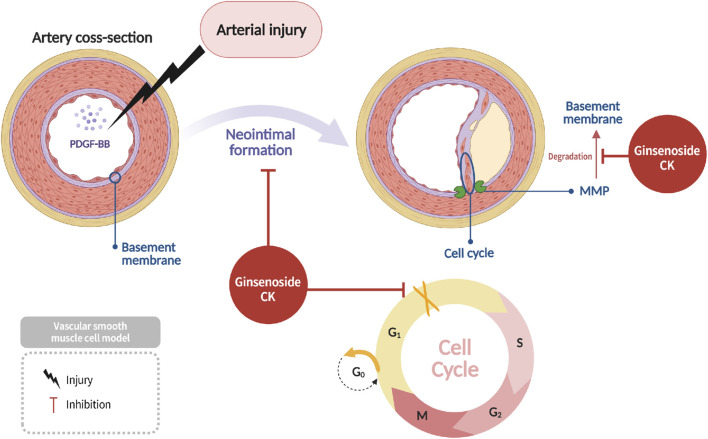
Suppression of PDGF-BB–induced vascular smooth muscle cell proliferation and intimal hyperplasia by CK through anti inflammatory and anti-migratory effects.

Vascular smooth muscle cells (VSMCs) are critical structural components of the vascular wall. In the event of vascular injury, platelets release platelet-derived growth factor-BB (PDGF-BB), which stimulates the proliferation and migration of VSMCs. However, excessive VSMC proliferation contributes to vascular stenosis and atherosclerosis. Research has demonstrated that ginsenoside CK suppresses PDGF-BB-induced VSMC proliferation by impeding the degradation of the vascular basement membrane via matrix metalloproteinases (MMPs), thus hindering VSMC migration into the intima (Park et al., 2013) (Figure 5). CK has also been shown to induce cell cycle arrest of VSMCs at the G1 phase, thereby reducing neointimal formation and ultimately decreasing the risk of vascular occlusion and rupture. This effect, therefore, confers a vascular protective role to CK (Lee et al., 2011) (Figure 5).

CK-induced inhibition of VSMC proliferation has been shown to be associated with modulation of *cyclin D1*, *CDK4*, and *p21* expression, leading to G1 cell-cycle arrest. Furthermore, CK has been shown to regulate matrix metalloproteinase activity by suppressing *ERK1/2* signaling, thus constraining extracellular matrix remodeling and intimal thickening.

### 2.5 Endothelial protection against oxidized LDL-induced damage

**FIGURE 6:**
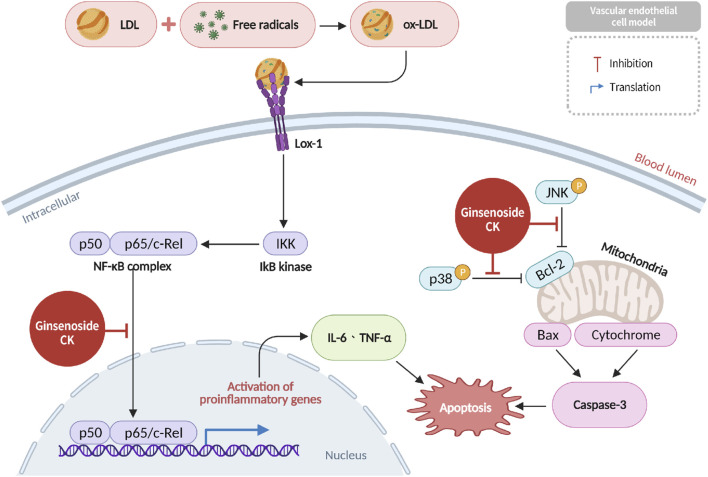
Protection of vascular endothelial cells by CK against oxidized LDL-induced inflammation and apoptosis via NF-κB downregulation.

Oxidized low-density lipoprotein (ox-LDL), a form of LDL modified by free radicals and strong oxidants, has been shown to injure vascular endothelial cells (VECs), inducing endothelial dysfunction, inflammation, and accelerating the development of vascular stiffness and atherosclerosis (Stocker and Keaney Jr, 2004). CK has been demonstrated to impede pivotal inflammatory signaling pathways, such as *NF-κB*, *p38 MAPK*, and *JNK*, in VECs. This process serves to attenuate ox-LDL-induced endothelial cell inflammation and apoptosis (see Figure 6 for further details). This protective action has been demonstrated to reduce endothelial dysfunction and the progression of atherosclerosis (Park et al., 2023).

### 2.6 Suppression of monocyte-endothelial interaction and CAM expression

**FIGURE 7:**
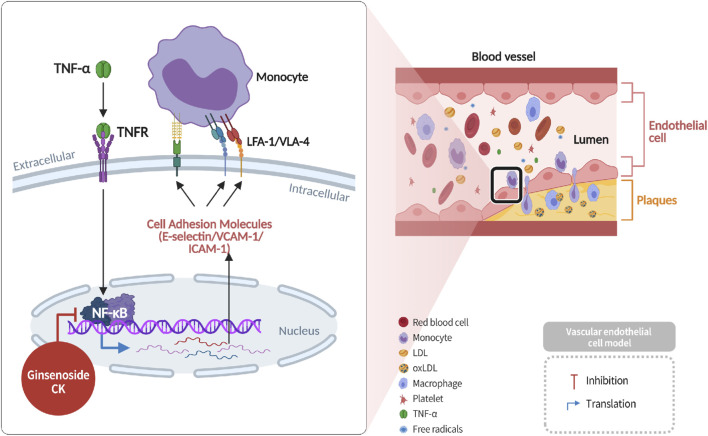
Inhibition of cell adhesion molecule expression and monocyte-endothelial adhesion by CK through suppression of the NF-κB pathway.

According to recent reports, ox-LDL has been shown to stimulate endothelial cells to express tumor necrosis factor-alpha (TNF-α) and upregulate cell adhesion molecules (CAMs), including E-selectin, vascular cell adhesion molecule-1 (*VCAM-1*), and intercellular adhesion molecule-1 (*ICAM-1*) (Hansson, 2005). These CAMs facilitate monocyte adhesion and transmigration into the vascular intima, where monocytes differentiate into macrophages and initiate inflammatory responses that contribute to endothelial dysfunction and plaque formation (Cybulsky and Gimbrone Jr, 1991). Ginsenoside CK has been demonstrated to impede *NF-κB*-mediated transcriptional activity, consequently diminishing the expression of E-selectin, *VCAM-1*, and *ICAM-1*, and hindering monocyte recruitment and atherogenesis (Lee et al., 2011) (Figure 7).

### 2.7 Neuroprotection against ischemic stroke via AMPK-mTOR modulation

**FIGURE 8:**
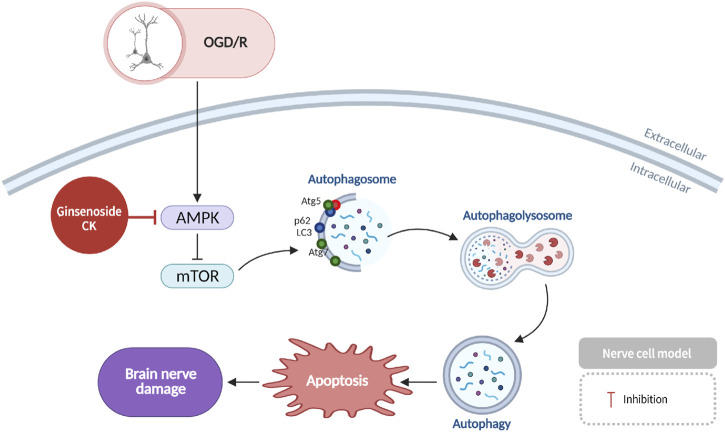
Neuroprotection by CK in ischemic stroke via regulation of AMPK–mTOR signaling and enhancement of energy metabolism.

AMP-activated protein kinase (AMPK) has been shown to play a critical role in energy homeostasis. In the context of ischemic stroke, the occlusion of cerebral vasculature results in the deprivation of neurons of oxygen and glucose, consequently inducing AMPK activation. This activation has been shown to inhibit mammalian target of rapamycin (*mTOR*) signaling and promote autophagy (Wang et al., 2018), which ultimately leads to neuronal death. In models of oxygen-glucose deprivation/reperfusion (OGD/R) that simulate ischemic stroke, CK has been documented to impede AMPK expression and neuronal apoptosis, consequently diminishing ischemic neuronal injury and manifesting neuroprotective effects (Huang et al., 2020) (Figure 8).

### 2.8 Ginsenoside CK protects against cerebral ischemia/reperfusion injury via Mul1/Mfn2-mediated mitochondrial dynamics and bioenergy

Cerebral ischemia/reperfusion (I/R) injury is a significant contributor to neuronal damage following stroke. The restoration of blood flow is imperative for reperfusion; however, this process paradoxically induces excessive oxidative stress, mitochondrial dysfunction, and energetic imbalance. One of the hallmark pathological features of I/R is the disruption of mitochondrial dynamics, particularly the imbalance between mitochondrial fusion and fission, which is imperative for maintaining neuronal viability (Peng et al., 2016; Ten and Galkin, 2019). In the presence of ischemia/reperfusion (I/R) conditions, the expression of the mitochondrial E3 ubiquitin ligase Mul1 is found to be significantly increased. Mul1 has been shown to promote the degradation of mitofusin 2 (Mfn2), a key fusion protein, thereby inhibiting mitochondrial fusion. Concurrently, Mul1 facilitates the post-translational modification and mitochondrial translocation of *DRP1*, a critical fission protein, enhancing mitochondrial fragmentation (Peng et al., 2016). The downregulation of *OPA1*, another fusion-related protein, further impairs the mitochondrial fusion process (Jin et al., 2021; Joaquim and Escobar-Henriques, 2020).

In the initial phase of reperfusion, mitophagy is sustained to a certain extent via the *PINK1/Parkin* pathway, which involves the ubiquitination of Mfn2, marking dysfunctional mitochondria for subsequent degradation. However, during prolonged I/R, the sustained decline in Mfn2 expression disrupts mitophagic clearance, leading to the accumulation of damaged mitochondria (Jin et al., 2021; Vranas et al., 2022). These dysfunctional mitochondria have been shown to increase reactive oxygen species (ROS) production through reverse electron transport, impair oxidative phosphorylation (OXPHOS), reduce ATP synthesis, and ultimately trigger apoptotic cascades through *BAX* oligomerization and cytochrome c release (Joaquim and Escobar-Henriques, 2020; Ten and Galkin, 2019).

**FIGURE 9:**
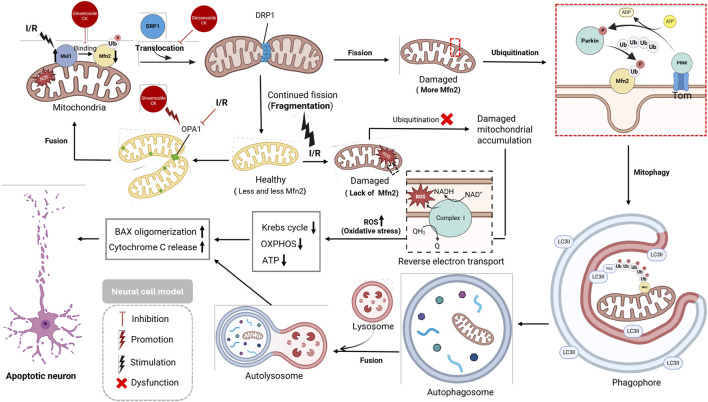
Preservation of mitochondrial dynamics during cerebral ischemia/reperfusion injury by CK through Mul1 inhibition and Mfn2 stabilization.

Recent studies have demonstrated that ginsenoside CK stabilizes mitochondrial dynamics by inhibiting Mul1-mediated Mfn2 degradation and reducing *DRP1* translocation. CK has also been observed to upregulate *OPA1* expression, thereby promoting mitochondrial fusion and structural integrity (Figure 9). The combined effect of these actions is the preservation of mitochondrial function, the reduction of ROS accumulation, the restoration of cellular energy homeostasis, and the protection of neurons from I/R-induced apoptosis (Jin et al., 2021; Joaquim and Escobar-Henriques, 2020; Peng et al., 2016; Ten and Galkin, 2019; Vranas et al., 2022).

### 2.9 Induction of neurogenesis after intracerebral hemorrhage via LXRα activation

**FIGURE 10:**
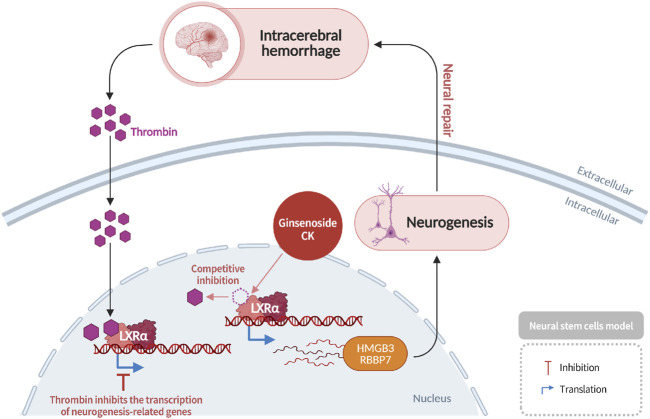
Promotion of neurogenesis following intracerebral hemorrhage by CK via activation of the LXRα–BDNF signaling pathway.

Intracerebral hemorrhage (ICH), a subtype of stroke caused by the rupture of cerebral blood vessels, results in impaired blood and nutrient delivery to brain tissue, leading to neuronal necrosis. Although less prevalent than ischemic stroke, ICH is associated with high mortality rates exceeding 30% (Qureshi et al., 2009). Chronic hypertension, a significant risk factor for ICH, has been demonstrated to promote vascular stiffening and fragility, thereby increasing susceptibility to rupture during periods of acute blood pressure spikes. Following the occurrence of an intracerebral hemorrhage (ICH), the subsequent activation of thrombin results in the initiation of downstream signaling cascades, which, in turn, promote neuronal and astrocyte death. This process ultimately leads to the development of cerebral edema, the disruption of the blood-brain barrier, and the manifestation of severe neurological deficits. Furthermore, thrombin has been observed to interact with nuclear liver X receptor alpha (*LXRα*), thereby inhibiting the transcription of genes associated with neurogenesis (Masada et al., 2001). Ginsenoside CK competitively binds to thrombin, thereby restoring *LXRα*-mediated transcription of neurogenic genes, such as *HMGB3* and *RBBP7*, and consequently enhancing neurogenesis and promoting functional brain repair (Zhou et al., 2020) (Figure 10).

### 2.10 Anti-inflammatory effects in microglia and stroke models

**FIGURE 11:**
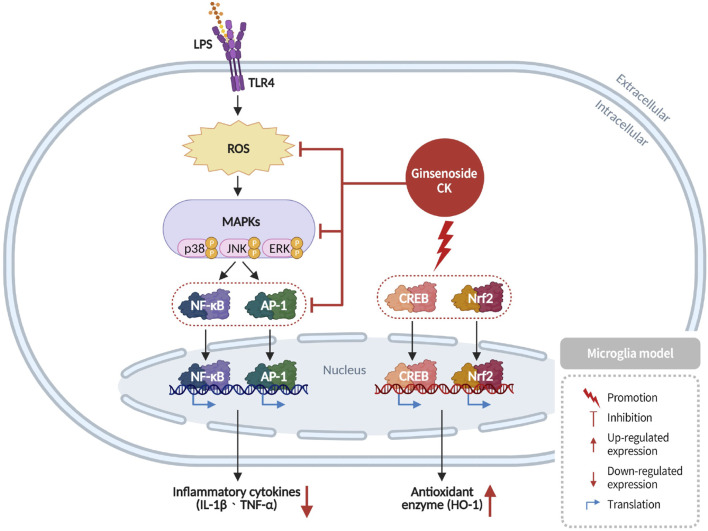
Anti-inflammatory effects of CK in stroke by inhibition of microglial activation and reduction of pro-inflammatory cytokine release.

Microglia, the resident immune cells of the central nervous system, fulfill a dual role in maintaining neuronal homeostasis and initiating immune responses. Upon pathogenic stimulation, microglia secrete inflammatory cytokines such as IL-1β and TNF-α to eliminate threats. However, sustained microglial activation leads to excessive cytokine release and neurotoxicity, establishing a vicious cycle of chronic neuroinflammation (Colonna and Butovsky, 2017). Ginsenoside CK has been shown to modulate microglial activation by enhancing antioxidant enzymes, such as heme oxygenase-1 (*HO-1*) and suppressing pro-inflammatory cytokine expression. This process has been demonstrated to mitigate neuroinflammatory damage and protect neuronal integrity (Park et al., 2012) (Figure 11).

## 3 Clinical potential of ginsenoside CK therapy for cardiovascular disease and stroke

Preliminary clinical and preclinical studies suggest that Ginsenoside CK is a promising therapeutic candidate for cardiovascular and cerebrovascular conditions. The following evidence has been cited to support this claim.

### 3.1 The following related compound clinical trial evidence has been cited

Evidence from structurally related compounds, Ginsenoside Rd—a structurally similar ginsenoside—demonstrated significant efficacy in a Phase II randomized controlled trial for acute ischemic stroke. This finding offers indirect evidence that supports the clinical potential of CK and related compounds (Kwon et al., 2022).

### 3.2 Preclinical evaluation of CK

Preclinical research indicates that CK possesses anti-inflammatory, endothelial protective, and neuroprotective properties in various animal models. Although human clinical trials of CK are pending, the results of this study provide a robust preclinical foundation for further investigation (Liu T. et al., 2022).

### 3.3 Synergistic effects with stem cell therapy

Recent evidence suggests that ginsenoside compound K (CK) enhances the angiogenic and neurovascular repair capacity of mesenchymal stem cells (MSCs) through activation of the *GLUT1* and *HIF-1α*/vascular endothelial growth factor (*VEGF*) signaling pathways. This synergistic interaction suggests a potential strategy to improve stem cell–mediated vascular regeneration following ischemic stroke.

## 4 Maximum tolerated dose and human safety of ginsenoside CK

A series of toxicological evaluations have yielded a high safety margin for ginsenoside CK. In rodent models, the single oral LD_50_ in mice exceeds 2000 mg/kg without acute toxicity, while long-term repeated dosing studies up to 26 weeks in rats at 100–180 mg/kg/day revealed no organ toxicity or biochemical abnormalities (Gao et al., 2019). In a similar vein, the chronic oral administration of 360 mg/kg/day to beagle dogs over a 26-week period did not result in any observed toxicity or adverse effects (Li et al., 2020).

### 4.1 Pharmacokinetic considerations and translational relevance

A primary challenge in the clinical translation of CK is the discrepancy between effective *in vitro* concentrations (5–50 μM) and the peak plasma levels (*C*_*max*_) observed in human Phase I trials (∼0.11–0.16 μM after a 100 mg dose). However, CK’s therapeutic relevance is supported by three key factors (Hu et al., 2023): Tissue accumulation: due to its high lipophilicity (LogP ≈5), CK preferentially accumulates in target organs. Preclinical data show heart and brain concentrations are 5- to 8-fold higher than in plasma, effectively reaching the micromolar threshold required for bioactivity (Morshed et al., 2024). Signal amplification: As an upstream modulator of *Nrf2* and *NF-κB*, CK acts as a signaling “tuner.” This allows it to trigger robust downstream cytoprotective cascades even at sub-micromolar systemic exposures (Kim, 2013; Oh and Kim, 2016; Chen et al., 2018). Chronic exposure: repetitive daily dosing results in a steady-state accumulation factor of 4- to 7-fold, further bridging the gap between acute PK profiles and long-term therapeutic efficacy (Jeong et al., 2025).

### 4.2 Enhancing clinical applicability via SMEDDS

In order to overcome the low oral bioavailability (∼12%–15%) and poor aqueous solubility of CK, Self-Microemulsifying Drug Delivery Systems (SMEDDS) offer a promising translational strategy. SMEDDS spontaneously form nano-emulsions (<100 nm) in the gastrointestinal tract, providing several advantages (Hu et al., 2023): Solubilization: This mechanism ensures the sustained availability of CK in a dissolved state, circumventing the limitations imposed by its rate of dissolution (Chen et al., 2018) (Morshed et al., 2024). Enhanced absorption: The augmented surface area of nano-droplets has been demonstrated to promote cellular uptake and facilitate lymphatic transport, thereby circumventing initial hepatic metabolic processes (Chen et al., 2018). Reduced variability SMEDDS has been demonstrated to minimize the “food effect,” thereby ensuring more consistent therapeutic levels across diverse patient populations. The integration of these nanocarrier technologies enables the optimization of systemic exposure of CK, thereby facilitating the attainment of the therapeutic windows required for the treatment of both acute and chronic cardiovascular conditions.

In extending these findings to the human context, clinical studies have demonstrated that ginsenoside CK is well tolerated in healthy volunteers. The administration of both single and multiple-dose regimens (ranging from 40 to 160 mg) did not result in any adverse events of significance, with serum biochemical parameters remaining within normal ranges. Notably, gender differences in pharmacokinetics were observed, with females showing slightly faster absorption; nevertheless, overall tolerability was excellent across all participants (Chen et al., 2018). The collective results of this study suggest that a maximum tolerated dose of ginsenoside CK exists, and that its long-term safety profile is robust (Table 3).

**TABLE 3:** Summary of acute, chronic, and human safety evaluations of Ginsenoside CK in preclinical and clinical studies.

Category	Model	Key findings	Dosage range	Conclusion
Acute toxicity	Rodents (mice, rats)	No significant toxicity observed	Single oral dose >2000 mg/kg	Safe
Chronic toxicity	Dogs (beagles)	No organ damage or blood abnormalities (26 weeks)	360 mg/kg/day	Well-tolerated
Human tolerability	Healthy volunteers	No major adverse reactions	40–160 mg (single dose)	Good tolerability

## 5 Discussion

The therapeutic potential of CK in cardiovascular and cerebrovascular protection is firmly supported by its diverse mechanistic pathways, ranging from antioxidant defense to anti-inflammatory modulation. However, bridging the gap between benchtop discovery and clinical application requires addressing two pivotal factors: standardized production and pharmacokinetic (PK) translation.

The practical acquisition of CK has historically been a bottleneck due to its scarcity in raw ginseng. The shift toward enzymatic biocatalysis and microbial transformation, as detailed in this review, ensures a scalable and high-purity supply. These biotechnological advancements provide the necessary chemical standardization (CMC) required for rigorous clinical evaluation, overcoming the limitations of traditional extraction.

Ginsenoside Compound K (CK) is a notable systemic bioactive metabolite derived from protopanaxadiol-type ginsenosides. It demonstrates superior membrane permeability and a multi-target pharmacological profile in comparison to its parent compounds. A pivotal mechanism elucidated in this review is CK’s sophisticated modulation of the *Nrf2* signaling pathway, the master regulator of cellular redox homeostasis. CK has been identified as a robust upstream signaling tuner. It has been demonstrated to promote *Nrf2* nuclear translocation and the subsequent activation of the Antioxidant Response Element (ARE), thereby orchestrating the transcription of a comprehensive suite of Phase II antioxidant enzymes, including heme oxygenase-1 (*HO-1*), superoxide dismutase (SOD), and glutathione peroxidase (GPx). The therapeutic relevance of this pathway is underscored by its ability to bridge the “concentration gap” between effective *in vitro* concentrations (5–50 μM) and the lower peak plasma levels (∼0.11–0.16 μM) typically observed in human Phase I trials. The CK-induced upregulation of *HO-1*, in particular, has been demonstrated to mediate potent anti-inflammatory effects by inhibiting microglial activation. The efficacy of CK in suppressing the release of pro-inflammatory cytokines, specifically IL-1β and TNF-α, is attributable to its ability to reinforce the *Nrf2*-ARE-*HO-1* axis. This, in turn, results in the interruption of the cycle of neuroinflammation and the preservation of neuronal integrity. Furthermore, this pathway provides sustained defense by generating protective metabolites, such as carbon monoxide and biliverdin, which synergistically mitigate mitochondrial ROS (mROS) generation and prevent the opening of the mitochondrial permeability transition pore (mPTP) during acute ischemia/reperfusion (I/R) injury. This transition from simple radical scavenging to a robust induction of endogenous cytoprotective and anti-inflammatory programs provides a plausible biological basis for CK’s efficacy despite its low systemic exposure. The implementation of SMEDDS offers a transformative translational strategy for the further optimization of these pharmacokinetic-pharmacodynamic relationships. SMEDDS have been shown to form spontaneous nano-emulsions that bypass dissolution-rate limitations and promote lymphatic transport. This property of SMEDDS has been demonstrated to significantly enhance CK’s oral bioavailability and reduce inter-patient variability. In summary, the diverse protective effects of CK, encompassing mitochondrial dynamics preservation and the suppression of neuroinflammatory cascades, suggest its potential for clinical translation in the treatment of complex cardiovascular and stroke-related pathologies.
